# PLASMA METABOLOMICS OF VIGOR TO FRAILTY AMONG OLDER BLACK AND WHITE MEN AND WOMEN

**DOI:** 10.1093/geroni/igad104.3741

**Published:** 2023-12-21

**Authors:** Megan Marron, Shanshan Yao, Ravi Shah, Venkatesh Murthy, Anne Newman

**Affiliations:** University of Pittsburgh, Pittsburgh, Pennsylvania, United States; University of Pittsburgh, Pittsburgh, Pennsylvania, United States; Vanderbilt University Medical Center, Nashville, Tennessee, United States; University of Michgan, Ann Arbor, Michigan, United States; University of Pittsburgh, Pittsburgh, Pennsylvania, United States

## Abstract

Older women and Black individuals are more likely to experience frailty. Metabolomics can provide a metabolic characterization of frailty that can help inform more effective interventions aimed at improving health, reducing disparities, and preventing frailty with aging. We sought to identify metabolites and pathways associated with vigor to frailty and determine whether these associations differed by gender and/or race among N=2189 older Black and White men and women from the Health, Aging, and Body Composition (Health ABC) study. Fasting plasma metabolites were measured using liquid chromatography-mass spectrometry. Vigor to frailty was based on weight change, physical activity, gait speed, grip strength, and energy level. We used linear regression of a single metabolite on vigor to frailty, adjusting for age, gender, race, study site, and multiple comparisons using a Bonferroni correction. Among 500 metabolites, 113 were associated with vigor to frailty (p< 0.0001). Associations between metabolites and vigor to frailty did not differ significantly by race and/or gender. Lower amino acids, glycerophospholipids, & sphingolipids and higher acylcarnitines, fatty acids, amino acid derivatives, carboximidic acids, benzenoids, nucleosides, organoheterocyclic compounds, carbohydrates, and trimethylamine oxide were associated with frailer scores. Pathway analyses identified the citrate cycle (p=0.00005), where higher levels of measured metabolites involved in the citrate cycle were associated with frailer scores. Daily calories and protein intake did not differ by vigor to frailty. Frailer Health ABC participants may have lower utilization of energy pathways, potentially a result of less efficient utilization of similar amounts of nutrients and energy demand than more vigorous participants.

